# Malignant Phyllodes Tumor of the Breast: A Practice Review

**DOI:** 10.3390/clinpract11020030

**Published:** 2021-04-06

**Authors:** Ângelo Bezerra de Souza Fede, Ronaldo Pereira Souza, Mauricio Doi, Marina De Brot, Cynthia Aparecida Bueno de Toledo Osorio, Guilherme Rocha Melo Gondim, Jose Claudio Casali-da-Rocha, Rima Jbili, Almir Galvao Vieira Bitencourt, Juliana Alves de Souza, Rafael Caparica Bitton, Fabiana Baroni Alves Makdissi, Solange Moraes Sanches

**Affiliations:** 1 Department of Medical Oncology, A.C.Camargo Cancer Center, São Paulo 01525-001, Brazil; ronaldo.souza@accamargo.org.br (R.P.S.); solange.sanches@accamargo.org.br (S.M.S.); 2 Department of Breast Surgery, A.C.Camargo Cancer Center, São Paulo 01525-001, Brazil; mauricio.doi@accamargo.org.br (M.D.); fabiana.makdissi@accamargo.org.br (F.B.A.M.); 3 Department of Anatomic Pathology A.C.Camargo Cancer Center, São Paulo 01525-001, Brazil; marina.debrot@accamargo.org.br (M.D.B.); cabtoledo@accamargo.org.br (C.A.B.d.T.O.); 4 Department of Radiation Oncology, A.C.Camargo Cancer Center, São Paulo 01525-001, Brazil; guilherme.gondim@accamargo.org.br; 5 Department of Oncogenetics, A.C.Camargo Cancer Center, São Paulo 01525-001, Brazil; casali.rocha@accamargo.org.br (J.C.C.-d.-R.); rima.jbili@accamargo.org.br (R.J.); 6 Department of Imaging, A.C.Camargo Cancer Center, São Paulo 01525-001, Brazil; almir.bitencourt@accamargo.org.br (A.G.V.B.); juliana.alves@accamargo.org.br (J.A.d.S.); 7 Institut Jules Bordet, Université Libre de Bruxelles (U.L.B.), 1050 Brussels, Belgium; rcaparica@hotmail.com

**Keywords:** Malignant phyllodes tumor, breast cancer, fibroepithelial neoplasm

## Abstract

Introduction: Phyllodes tumor (PT) of the breast, particularly malignant phyllodes tumor (mPT), is a rare fibroepithelial neoplasm. A complex diagnosis is based on pathologic, radiologic, and clinical findings, with controversies about what is the best therapeutic strategy. Objective: Our objective was to provide an overview of the clinical, pathologic, and therapeutic aspects of this rare tumor. Conclusions: mPT is a rare presentation of breast cancer and a challenge in clinical practice. A multidisciplinary approach should take into account some aspects like pathogenic mutations and hereditary syndromes. Oncologic surgery is the fundamental approach, and the use of adjuvant therapies is still controversial due to the lack of clinical trials. Treatment recommendations should be individualized according to patient risk and preferences. Prospective studies are fundamental to clarifying the best treatment for these tumors.

## 1. Introduction

Phyllodes tumor (PT) of the breast is a rare fibroepithelial neoplasm, representing 0.3 to 1% of all breast tumors [1]. Such cases are classified into benign, borderline, and malignant (mPT) according to a combination of several histologic features. The rarity of these lesions, particularly malignant PTs, contributes to the challenge in defining the most appropriate approach for these patients. Additionally, there is a difficulty in differentiating malignant PT from other types of rare tumors, and most treatments are based on retrospective studies and small series of patients, showing the importance of discussing this theme. In this review we explore the main aspects of mPT, its clinical presentation, and possible treatments based on the most recent literature.

## 2. Phyllodes Tumors—Pathologic Features

Phyllodes tumors (PTs) are biphasic neoplasms that have in common the presence of a leaf-like architecture resulting from a prominent intracanalicular growth pattern, cleft-like spaces lined by epithelial and myoepithelial cells, and hypercellular stroma. Frond-like projections of cellular stroma lined by epithelium generate the peculiar leaf-like appearance. The epithelial component may show apocrine or squamous metaplasia and usual ductal hyperplasia [1,2]. Lobular neoplasia, atypical ductal hyperplasia, ductal carcinoma in situ, and invasive carcinoma may also be found within the lesion, although very rarely [1,2,3]. PTs exhibit a broad spectrum of morphologic characteristics; hence, strict histologic evaluation of a combination of morphologic criteria helps in achieving an accurate diagnosis and providing valuable clinical information [1,2,3].

Upon gross examination, PTs form circumscribed and firm masses with a tan or pink to grey-colored cut surface, which may be fleshy or mucoid. The typical whorled pattern with curved clefts and cystic spaces may be evident, particularly in larger lesions. Foci of hemorrhage and necrosis may also be found [1,2].

**Figure 1:**
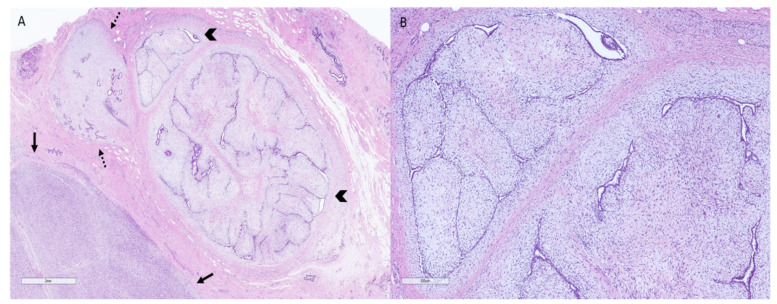
Surgical specimen of a patient diagnosed with a malignant phyllodes tumor displaying areas with benign, borderline, and malignant features. (**A**): Representative micrograph of the excision specimen showing a heterogeneous lesion with areas of marked (arrows), moderate (dotted arrows), and mild (arrowheads) stromal cellularity (hematoxylin–eosin, original magnification 10×). (**B**): Representative micrograph of an area of the same neoplasm and specimen exhibiting an intracanalicular growth pattern, low stromal cellularity, and no cytologic atypia (hematoxylin–eosin, original magnification 50×).

Phyllodes tumors are classified as benign, borderline, or malignant based on histologic characteristics. According to the latest edition of the World Health Organization (WHO) Classification of Breast Tumors (2019), morphologic criteria to grade these lesions include tumor border, stromal cellularity, stromal cell atypia, stromal cell mitotic activity, presence of stromal overgrowth, and presence of malignant heterologous elements. Assessment of a few of these features can be somewhat subjective, although the distinction between benign and malignant PT (mPT) is usually straightforward. However, for borderline PTs, the diagnostic criteria are not as clear-cut. It is noteworthy that the identification of malignant heterologous elements is sufficient for a diagnosis of mPT even if other parameters of malignancy are not seen, with the exception of well-differentiated liposarcoma. Furthermore, PTs may show areas with benign, borderline, and malignant features intermixed within the same neoplasm, making diligent gross examination and histologic sampling most relevant (Figure 1) [1,2,3].

**Figure 2:**
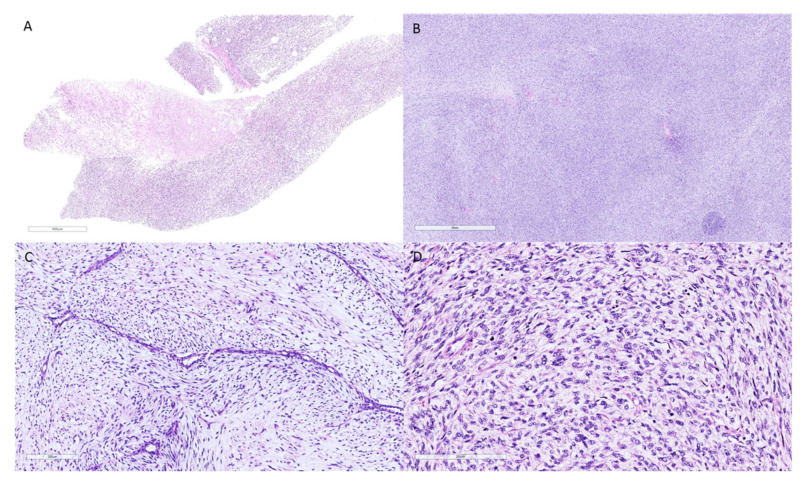
Photomicrographs of the core needle biopsy and surgical excision of a patient initially diagnosed with a malignant spindle cell neoplasm of the breast. (**A**): Core needle biopsy of a malignant spindle cell neoplasm without evident epithelial elements (hematoxylin–eosin, original magnification 10×). (**B**–**D**): Surgical excision of the lesion confirmed the diagnosis of malignant phyllodes tumor, as the presence of a benign epithelial component was demonstrated. (**B**): Tumor areas with diffuse stromal overgrowth and high stromal cellularity (hematoxylin–eosin, original magnification 20×). (**C**): Cleft-like spaces covered by benign epithelium with sub-epithelial stromal condensation (hematoxylin–eosin, original magnification 200×). (**D**): High magnification of the neoplasm exhibiting a markedly cellular stroma, pleomorphic stromal cells, and numerous mitoses (hematoxylin–eosin, original magnification 200×).

Malignant PTs are characterized by the presence of the following parameters (Figure 2): stromal overgrowth, as defined by the identification of at least one low-power microscopic field (40× magnification: 4× objective and 10× eyepiece) containing only stroma without associated epithelial elements, marked stromal nuclear pleomorphism, prominent mitotic activity (≥5 mitoses/mm^2^ or ≥0 mitoses per 10 high-power fields of 0.5 mm^2^), increased stromal cellularity, and usually marked and diffuse, permeative, or infiltrative borders. Heterologous differentiation may be encountered in the form of chondrosarcoma, osteosarcoma, liposarcoma, and rhabdomyosarcoma. Due to extensive sarcomatous overgrowth, demonstration of residual epithelial structures is sometimes challenging, and a comprehensive sampling can be necessary to reach a correct diagnosis. Additionally, these lesions must be distinguished from metaplastic carcinomas and sarcomas (primary or metastatic), the latter being extremely rare in the breast. Still, the clinical outcomes of primary breast sarcomas and malignant PTs seemingly tend to be comparable [1,2,3].

Metaplastic carcinomas can present as a malignant spindle cell neoplasm with or without heterologous elements, similar to malignant PTs. The observation of a leaf-like pattern and benign epithelium covering cleft-like spaces is classic of PTs, while the identification of malignant epithelial elements favors metaplastic carcinoma [1,2]. If there is no morphologic evidence of an epithelial component, especially on core needle biopsies (Figure 2), immunohistochemistry with a panel of cytokeratins (CKs; MNF116, AE1/AE3, CK5/6, CK14, 34bE12, CAM 5.2, and CK7) and myoepithelial cell markers (*p*63) to confirm an epithelial histogenesis may be required [4,5] Most PTs are negative for CKs and *p*63, whereas expression of CD34 is detected in 37% to 57% of cases. In contrast, metaplastic carcinomas are negative for CD34 [1,2,3]. Nevertheless, previous studies have also reported very focal positivity for CKs and *p*63 in malignant PTs, suggesting caution in classifying malignant spindle cell tumors of the breast on core biopsy [4,5].

## 3. Phyllodes Tumors—Pathogenesis and Molecular Findings

The molecular features of PT are still poorly defined, and a deeper understanding of the genetics of these tumors may help us to understand their pathogenesis and progression and to potentially identify novel treatment approaches.

Epithelial–stromal interactions are implicated in the pathogenesis of PTs and originate from both the intralobular and periductal stroma. When the stroma becomes independent of epithelial interactions, malignant transformation and autonomous stromal growth take place [1,2].

Breast fibroepithelial lesions are underpinned by recurrent MED12 exon 2 somatic mutations in stromal cells (59–67% of fibroadenomas and 45–67% of PTs), particularly in fibroadenomas and benign PTs. In fact, MDM2 mutations seem to be an early founder event in the pathogenesis of both entities [1,2,6,7]. In contrast, whereas fibroadenomas lack copy number changes, most PT show chromosomal instability, such as recurrent loss on chromosome 1q, 4p, 10, 13q, 15q, 16, 17p, 19, and X, involving loss of loci of TP53 and *CDH1*, and recurrent copy number gains on 1q, 2p, 3q, 7p, 8q, 16q, and 20 [8].

Many chromosomal imbalances have been reported in PTs by array comparative genomic hybridization (array-CGH), such as frequent gains at 1q, 5p, 7, and 8 and losses at 6, 9p, 10p, and 13, with an increasing rate of genetic defects from benign to malignant tumors [9]. Also, interstitial deletions of 9p21 that involved the CDKN2A locus were found to be present in many malignant/borderline PTs, and some of these appeared to cause a second hit, such as frequent homozygous loss, but also CDKN2A point mutation and methylation [9]. The intra-tumoral genetic heterogeneity based on the number of chromosomal instabilities was reported to be greater in the borderline than in the malignant compartment of a giant bilateral PT [10].

MED12 exon 2 and TERT mutations are the most frequent alterations found in PT, corresponding to 70% of cases, and although their frequencies increase from benign to malignant, they are not useful in distinguishing between PT subtypes [11]. A number of somatic variants might be useful to clarify the molecular characteristics of PT, especially distinguishing borderline and malignant PT. Mutations in PIK3CA, RB1, TP53, NF1, ERBB4 and EGFR have been reported in malignant PT, and they might promote the progression of borderline to malignant PT [12]. Activating mutations in EGFR and the overexpression of EGFR were associated with the progression in grade of PT [13].

Genomic profiling of PTs revealed aberrations in FGFR1 and PI-3 kinase/RAS signaling pathways in 80% of malignant PTs, including activating hotspot mutations in FGFR1 identified in 2 out of 10 malignant PTs (2/10), in the TERT promoter (6/10), TP53 (4/10), PIK3CA (3/10), MED12 (3/10), SETD2 (2/10), and KMT2D (2/10). Actionable activating FGFR1, PIK3CA, and BRAF V600E mutations, inactivating TSC2 mutation, EGFR amplification, and PTEN loss represent potential targets for precision oncology in advanced PT [14].

A targeted deep sequencing on 17 PTs, including 13 malignant PTs, aimed to identify the associations between genetic alterations and clinical prognosis. As expected, the most frequently detected genetic alteration occurred in the TERT promoter region (70.6%), followed by MED12 (64.7%). Interestingly, EGFR amplification and TP53 alteration were detected in four malignant PTs without genetic alterations in the MED12 and TERT promoter regions, suggesting different progression pathways. RARA and ZNF703 mutations were associated with local recurrence; SETD2, BRCA2, and TSC1 were detected in PTs with distant metastasis; and PTEN and RB1 copy number deletion showed rapid disease progression in malignant PTs [15].

Finally, another study defined the landscape of PT for actionability. In addition to the common TERT and MED12 mutations, malignant PT harbored loss-of-function mutations in TP53, while deleterious mutations in the tumor suppressors RB1 and NF1 were identified exclusively in malignant tumors. High-level copy-number alterations (CNAs) were nearly exclusively confined to malignant PTs, including potentially clinically actionable gene amplifications in IGF1R and EGFR [16].

## 4. Hereditary Genetic Features

PT has been associated with Li–Fraumeni syndrome (LFS), a rare autosomal dominant syndrome related to pathogenic or likely pathogenic variants (PVs) in the TP53 gene, predisposing the carrier to a broad spectrum of tumors throughout life. It was initially described in 1969 by Frederick Li and Joseph Fraumeni in association with sarcoma and other adult malignancies. Clinical suspicion of LFS can be founded on the occurrence of typical tumors at defined ages of diagnosis for affected members in a suspected family. The diagnosis of LFS is established in a proband who meets all three classic LFS criteria and/or has a germline PV in TP53 identified by molecular genetic testing [17,18].

The p53 protein has many cellular functions and plays a central role in genome integrity. Germline TP53 PVs are well distributed all across the coding regions of the gene, and even simple amino acid changes (missense variants) might cause serious damage to p53 transcription activities. Usually, germline PVs in heterozygosity in the TP53 gene have been mostly associated with the classical clinical spectrum of LFS, including PTs of the breast, since its first description by Birch et al. in 2001 [19].

In Latin countries like Brazil, especially for the states in the South and Southeast regions, a highly prevalent and moderate-risk PV, the missense TP53 R337H, has been studied in a number of tumor types of the LFS clinical spectrum, including PTs. The prevalence of the TP53 R337H was analyzed in 148 PTs of the breast from archived formalin-fixed, paraffin-embedded (FFPE) blocks. The R337H variant was identified in eight cases, equivalent to 5.4%. The mutation frequency was significantly higher among malignant tumors (3 of 13 tumors; 23%) than among benign tumors (5 of 128 tumors; 3.4%) (*p* = 0.004) [20].

Recently, Pinto and collaborators described two distinct haplotypes in carriers of R337H which can be distinguished by the presence or absence of the XAF1 E134* co-variant, a non-sense mutation that appears to modulate the functional activity of the p53 protein [21]. This might have direct implications in the genetic counseling of carriers of R337H in the near future. PT has been occasionally described in association with other hereditary cancer syndromes, such as hereditary breast and ovarian cancer (HBOC, PV in BRCA1), hereditary retinoblastoma (PV in RB1), and Lynch syndrome (PV not reported in MSH6), mostly as case reports [22,23,24]. Somatic mutation analysis of PTs may highlight the molecular pathways and candidate genes involved in their carcinogenesis. A recent Polish study evaluated a hotspot gene somatic panel in 10 paired primary and metastasis malignant PTs and detected four PVs: three in the CDKN2A gene (one of the cases had another PV in the TP53 gene) and one PV in the PTEN gene; the authors believed that the CDKN2A gene might be involved in the development of PT of the breast, as well as its recurrence and metastasis [25].

In the largest collaborative study, which involved 11 institutions and 550 women with PTs, 59.8% of them reported a significant family history of cancer, and 34% reported more than three affected relatives. Genetic testing was performed in a minority, only 6.2% of them (or 34 women), and consisted in the Next Generation Sequencing (NGS) analysis of a 31 multigene cancer panel; in 13 cases, it was limited to only the BRCA1 and BRCA2 genes. The mutation detection rate was 8.8%: one PV was found in BRCA1 and two PVs in TP53. Among the 21 women who were tested for TP53, 9.5% showed a PV [26]. The authors attributed this undertesting to multiple factors, including that (1) National Comprehensive Cancer Network (NCCN) criteria do not include PT as a specific criterion for any Genetic/Familial High Risk Assessment categories; (2) genetic counselors do not consider PT as a soft tissue sarcoma, precluding inclusion within the sarcoma criteria; (3) providers are unaware of the association between TP53 and PT; and (4) the associations with various other germline mutations and PT are currently unknown, leading providers to potentially overlook standard indications for genetic referral and evaluation [26].

There is little information regarding the association of PT with the presence of germline mutations in genes of hereditary predisposition to cancer. The moderate-risk variant R337H in the TP53 gene could play an important role in the pathogenesis of PT in Brazil, especially in malignant phyllodes and with the presence of the XAF1 E1344* co-mutation. Further large, multicentric, translational studies are necessary to better understand and molecularly characterize PT of the breast.

## 5. Imaging Findings

**Figure 3:**
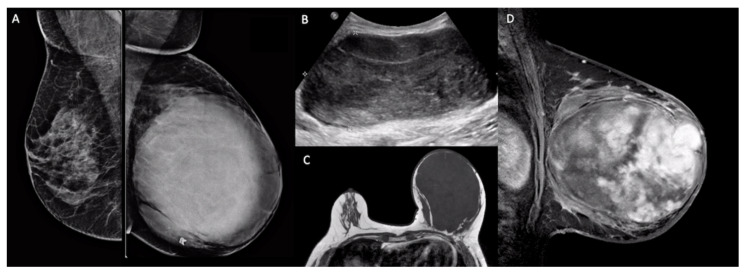
Imaging findings of a malignant phyllodes tumor. (**A**): Bilateral mammography (mediolateral oblique view) showed a large, round, circumscribed, high-density mass in the left breast. (**B**): Breast ultrasound showed an oval, parallel, circumscribed mass with a heterogeneous echo pattern. (**C**): T1-weighted axial magnetic resonance imaging (MRI) showed a large, hypointense, circumscribed mass in the left breast. (**D**): T1-weighted sagittal fat-saturated contrast-enhanced MRI showed heterogeneous internal enhancement within the mass.

Phyllodes tumors have image characteristics similar to those of fibroadenomas. They usually present as an oval, round, or lobulated circumscribed mass, with rapid growth and large dimensions in mammography, ultrasound, and magnetic resonance imaging (MRI) (Figure 3). Ultrasound may show hypoechoic, heterogeneous, or complex cystic and solid echo patterns. MRI usually shows heterogeneous internal enhancement due to the presence of cysts, necrosis, or septations, which may be related to the tumor’s rapid growth [27]. Imaging can be used for differential diagnosis with other breast masses, for locoregional staging, and to guide percutaneous biopsies.

Benign, intermediate, and malignant phyllodes breast tumors have similar imaging features; however, some MRI findings can be used to help determine the risk of malignancy. Non-circumscribed margins, cystic components, irregular cyst walls, peritumoral edema, low signal intensity on T2-weighted images, and low apparent diffusion coefficient (ADC) are correlated to higher histologic grade, presence of stromal hypercellularity, hemorrhagic infarction, and necrosis on histopathology [28,29].

## 6. Clinical Findings

The majority of phyllodes tumors occur in women, with a median age of presentation of 45 years [30]. PTs are usually identified as a breast nodule or mass in a physical or radiological exam. Patients usually have a well-defined, firm nodule or mass that is mobile and painless. Tumor size is variable, from small to occupying the entire breast (average 4 to 7 cm). Large tumors may infiltrate the skin and tend to grow faster when compared to fibroadenomas, their main point of difference in diagnosis [30,31,32].

Phyllodes tumors are benign in the majority of cases, and malignant PTs comprise 10% to 15% of cases [1,2,3]. An overall rate of local recurrence of 21% has been demonstrated, with ranges of 10–17%, 14–25%, and 23–30% for benign, borderline, and malignant PTs, respectively. Relapses usually occur within 2–3 years of diagnosis and are of a higher grade compared to the original neoplasm in 31.5% of cases. Amongst the predictors of recurrence are margin status, stromal overgrowth, stromal atypia, and mitotic activity [2].

Axillary lymph node metastases are uncommon, while distant metastases affect only 2% of patients in general—nearly entirely those diagnosed with malignant PTs—within 5–8 years of diagnosis. Large tumor size and malignant heterologous elements are related to a higher risk of distant relapse [1,2]. About 9% to 28% of malignant PTs progress with distant metastases [1,3], mainly to the lung and skeleton [2,3]. Patients with metastasis usually do not respond to chemotherapy and have poor survival in most cases [1]. It is difficult to predict patient outcomes, and a normogram has been developed and validated to estimate clinical behavior. The so-called Singapore Normogram takes into consideration three histological criteria (atypia, mitoses, and overgrowth) and surgical margin status to calculate the recurrence-free survival of an individual woman diagnosed with PT [2,33].

## 7. Surgical Treatment

Based on the NCCN guidelines, the surgical treatment for malignant phyllodes tumors is a complete surgical excision with 1 cm margins without axillary surgery. The low incidence of lymph node metastasis in previous studies supports the recommendation to not perform axillary surgery in these cases [32,34,35].

There is some evidence that surgical margins impact the risk of relapse. In a previous study with 48 cases of high-grade mPT, wide local excision with margins greater than 1 cm resulted in fewer local recurrences (60% versus 28%) within a follow-up period of 9 years in comparison with narrow margins [35]. Finally, in a systematic review and meta-analyses of 9234 individual cases, 18% of these patients having malignant phyllodes tumors, a positive surgical margin was significantly associated with a higher local recurrence risk (OR 6.85; 95% CI 1.58–29.64) [36].

Mastectomy at the index surgery is only recommended in cases with an inability to adequately obtain 1 cm margins or if cosmetic changes to the breast would be unacceptable to the patient [32]. These recommendations are based in previous studies where mastectomy was not found to provide a benefit in terms of cancer-specific mortality versus wide excision [34].

## 8. Adjuvant Radiotherapy in Malignant Phyllodes Tumors

Even when mPT is resected with free margins, local recurrence and distant metastases occur in some cases [37]. Retrospective studies and a single small prospective study suggest the benefits of radiotherapy (RT) in local control after breast-conserving surgery (BCS).

A population analysis showed that adjuvant RT reduced the risk of local recurrence in mPT by 57%, and that the greatest benefit occurred after BCS [38]. This same study demonstrated that the indication for adjuvant RT has increased by 100% in the last 10 years, being currently indicated in 20% of patients with mPT [39].

A Chinese meta-analysis of six non-randomized retrospective studies with a total of 2058 patients demonstrated that RT after BCS reduced the risk of local recurrence by 69%, but not after mastectomy [40]. In a prospective multi-institutional study with 46 patients (30 mPT and 16 borderline), no local recurrence was observed in 10 years after BCS with free margins and adjuvant RT, but no improvement in overall survival was observed with adjuvant RT [38].

## 9. Chemotherapy in Early Stage and Metastatic Disease

The role of complementary adjuvant therapies such as chemotherapy and radiotherapy after surgery with free margins is controversial [41,42]. While the risk of distant recurrence can reach figures above 30%, a prospective series failed to demonstrate any benefit in overall survival or reduction in the risk of distant recurrence with adjuvant chemotherapy (ChT) [43]. On the other hand, these series have serious limitations, including a small number of studies and patients involved, non-randomized series, a long recruitment period, and lack of clarity in stratification according to the risk of clinical and pathological recurrence [44].

In the largest prospective and observational study conducted, involving 28 patients and requiring 10 years for recruitment, with a median follow-up of 15 months (range, 2–81 months), doxorubicin and dacarbazine did not improve recurrence-free survival (RFS) when compared to observation, with a tendency of inferiority to adjuvant chemotherapy. The five-year RFS rate was 58% (95% CI = 36% to 92%) for the patients who received adjuvant therapy and 86% (95% CI = 63% to 100%) for the patients who did not (*p* = 0.17) [44]. In a Japanese retrospective series with 70 patients, although 51 were low-grade or borderline and only 8 received adjuvant chemotherapy, in univariate analysis, adjuvant chemotherapy was not associated with a better disease-free survival (DFS) or overall survival (OS) rate [45]. In the face of limited current evidence, patients with mPTs should be encouraged to participate in clinical trials to determine the best adjuvant strategy.

In view of the controversial issues, the discussion of chemotherapy involves the assessment of the individual risk factors of distance recurrence, such as tumor size, stromal overgrowth, and positive surgical margin, in case of impossibility of re-excision surgery [41,46,47,48,49,50,51]. A previous trial evaluated 101 patients, including 30 mPTs, and found the stromal overgrowth to be the only independent predictor of distant failure. The 5-year and 10-year survival rates for patients with and without stromal overgrowth were 81% and 42% and 92% and 79%, respectively (*p* = 0.0115). Of those tumors with stromal overgrowth and size greater than 5 cm in greatest dimension, 6 of 14 patients (43%) developed distant metastasis [52]. According to the MD Anderson Cancer Center Clinical Practice Algorithm, tumors larger than 5 cm and with the presence of stromal overgrowth could be treated as stage III extremity sarcomas [53]. The European Society for Medical Oncology (ESMO) guideline considers using adjuvant criteria similar to those of extremity sarcomas [50].

Given the conflicting results, adjuvant chemotherapy is not the standard treatment in mPT. In the absence of clinical trials, it can be proposed as an option for high-risk individual patients (>5 cm tumor, stromal overgrowth, and positive surgical margin when there is no possibility of re-excision surgery) for shared decision-making with the patient. The treatment suggestion is the use of protocols based in anthracyclines and alkylating agents for three cycles.

In the metastatic scenario, the prognosis is adverse. The median overall survival varies between 5 and 17 months [46,47]. In the presence of neurological symptoms, brain metastasis should be investigated [49,54]. As in the adjuvant setting, metastatic patients should participate in clinical trials, if possible. In first-line therapy, anthracycline and ifosfamide (AI) in combination may be more effective in patients who have performance status for combination therapy. Series of cases have shown that the AI combination has a response rate of 53% and results in a median progression-free survival of 9 months, which shows a tendency for superiority over ifosfamide monotherapy and is significantly higher when compared to other chemotherapy protocols [52]. After progression, in addition to a new line of chemotherapy, pazopanib or trabectidine are reasonable options [32,53].

## 10. Conclusions

mPTs are rare neoplasms of the breast that are a challenge in clinical practice. An optimal diagnosis is reached based on a group of pathologic, genetic, and radiologic patterns. Some aspects should be taken into account, like genetic counseling, searching for pathogenic germline mutations (like TP53) with emphasis on Li–Fraumeni syndrome. The best approach after the diagnosis of mPT is based on a multidisciplinary discussion, with the surgical approach being the main therapy related to reducing the risk of disease recurrence. Adjuvant therapies with chemotherapy and radiotherapy are controversial and based on the results of clinical studies with a small number of patients, making a standard recommendation difficult. An international consortium of large cancer centers with clinical experience in the management of mPT should be encouraged and could be an important source of evidence for recommending a standardized treatment. In conclusion, in view of the rarity of this neoplasia, efforts are essential to better understand the disease, and the development of prospective studies could clarify the best approach for these patients.

## Data Availability

No new data were created or analyzed in this study.
